# Supplementary material for: Maternal Neutrophil Depletion Fails to Avert Systemic Lipopolysaccharide-Induced Early Pregnancy Defects in Mice
Source: Int J Mol Sci. 2021 Jul 25;22(15):7932. doi: 10.3390/ijms22157932 (PMC8347248; doi:10.3390/ijms22157932)
Supplement: Supplementary file 1 [file ijms-22-07932-s001.zip › ijms-1266489-SI.pdf]

Supplementary Materials

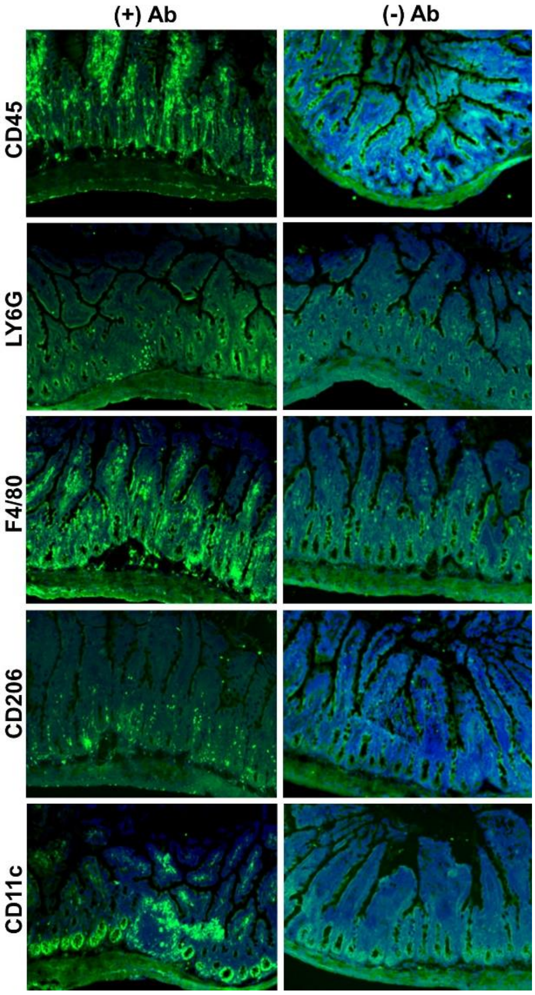

Figure S1. Immunofluorescence analyses ascertained specificity of primary and secondary antibodies in the intestine. Cryosections from this tissue were fixed and incubated with (+) or without (-) primary antibodies (Abs) prior to addition of secondary antibodies tagged with Alexafluor 488 (green) to check specificity of the primary and the secondary Abs. Total leukocytes and leukocyte subtypes were identified using Abs against CD45, Ly6G, F4/80, CD206, CD11c antigens and DAPI (blue) for nuclear staining.

Table S1. List of antibodies used in immunofluorescence studies.

Antibodies	Host	Source	Catalog #	Dilution/ Fixative
		**Primary antibodies**		
CD45	Rat	BD Pharmingen, Franklin Lakes, NJ, USA	550539	1:50/Ace
Ly-6-G	Rat	BioXCell, Lebanon, NH, USA	BP0075-1	1:100/ 10% BFP
Ly-6-C	Rat	Biolegend, San Diego, CA, USA	128001	1:100/ 10% BFP
F4/80	Rat	Abcam, Cambridge, MA, USA	ab6640	1:100/ Ace
CD206	Rat	Biolegend, San Diego, CA, USA	141709	1:100/ Ace:Met (1:1)
CD11c	Hamster	Novus Biologicals, Littleton, CO, USA	NBP1-06651	1:400/

				4% PFA
**Secondary antibodies**				
Goat anti-rat Alexa Fluor 488	Goat	Abcam, Cambridge, MA, USA	A-11006	1:1000
Goat anti-rat Alexa Fluor 555	Goat	Abcam, Cambridge, MA, USA	A-21434	1:1000
Goat anti - Hamster Alexa Fluor 488	Goat	Jackson Immuno Research Laboratories Inc, West Grove, PA, USA	127-545-160	1:150

Ace: Acetone; BFP: Buffered Formalin Phosphate; Met: Methanol; PFA: Paraformaldehyde.

Table S2. Primer sequence for quantitative RT-PCR.

Genes	Primer (5'-3')<br>S= Sense, AS=Anti-sense
GAPDH	S: CGACTTCAACAGCAACTCCCACTCTTCC<br>AS: TGGGTGGTCCAGGGTTTCTTACTCCTT
Cxcl1	S: ACTGCACCCAAACCGAAGTC<br>AS: TGTCAGAAGCCAGCGTTCAC
Cxcl2	S: CACCAACCACCAGGCTACAG<br>AS: GCTCCTCCTTTCCAGGTCAG
MyD88	S: ACTGAAGGAGCTGAAGTCGC<br>AS: AGTTCCGGCGTTTGTCCTAG
Trif	S: TGAGGAGCCTCCAGACTTGT<br>AS: CCAGTCTGGTGTGTCAATGG
Tnfα	S: GCCTCTTCTCATTCCTGCTTG<br>AS: CTGATGAGAGGGAGGCCATT
Il1β	S: CTGTGTCTTTCCCGTGGACC<br>AS: CAGCTCATATGGGTCCGACA
Il6	S: AACGATGATGCACTTGCAGA<br>AS: GAGCATTGGAAATTGGGGTA
Ifnβ	S: GCACTGGGTGGAATGAGACT<br>AS: AGTGGAGAGCAGTTGAGGACA
